# Platinum Amalgam

**Published:** 1872-11

**Authors:** 

**Affiliations:** in Missouri Dental Journal.


### ARTICLE VII.

### *Platinum Amalgam.*

"This alloy, if mixed with a small quantity of mercury, sufficient only to make it adhesive under strong pressure, sets in about ten minutes, sufficiently hard to be condensed in the same manner as a gold filling. It is then sufficiently hard for mastication, and is *absolutely free from shrinkage or alteration*, retaining its color and finish in some cases for years without the slightest change. It is totally insoluble, and possesses every desirable property of a perfect plastic metallic filling.

"The time required to harden may be adjusted at will by a slight variation in the proportion of mercury used. After the amalgam has become hard, the mercury cannot be separated under a dull red heat.

"All waste should be put in the bench sweep or gold fillings for refining, as it contains a large per centage of both gold and platinum.

"In 1oz., ½oz., and ⅛oz. (*Sample*) bottles 20 dollars per oz.

"Prepared and tested by Thos. Fletcher, F. C. S., 15 Bold street, Warrington."

[Having received the above advertisement by circular, and supposing that many other members of the profession have also, I have it inserted here for the purpose of remarking that I consider the gold and platinum, which is combined in this amalgam, of no positive benefit to it. Gold amalgam and platinum amalgam have been tried, without finding any superiority by the use of these expensive metals. Experience alone can decide upon the merits of any amalgam, and a new one should be used with caution. As for myself, I use none of them—*Dr. Chase, in Missouri Dental Journal.*

